# Ghost cell odontogenic carcinoma with suspected cholesterol granuloma of the maxillary sinus in a patient treated with combined modality therapy: A case report and the review of literature

**DOI:** 10.1097/MD.0000000000009816

**Published:** 2018-02-16

**Authors:** You Qin, Yanwei Lu, Liduan Zheng, Hong Liu

**Affiliations:** a Cancer Center; b Department of Pathology; c Department of Anesthesiology, Union Hospital, Tongji Medical College, Huazhong University of Science and Technology, Wuhan, China.

**Keywords:** calcifying epithelial odontogenic tumor, cholesterol granuloma of the maxillary sinus, combined modality therapy, ghost cell odontogenic carcinoma, odontogenic tumor

## Abstract

**Rationale:**

Ghost cell odontogenic carcinoma (GCOC) is a rare malignant odontogenic tumor with aggressive growth characteristics.

**Patient concerns:**

A 41-year-old Chinese male visited our hospital in 2013, with a 4-month history of bloody purulent rhinorrhea with a peculiar smell in the right nasal cavity.

**Diagnoses:**

The patient suffered from recurrent GCOC with suspected cholesterol granuloma of the maxillary sinus. The patient was incorrectly diagnosed with a calcifying epithelial odontogenic tumor at his first recurrence. Physical examination, radiological examination, and histopathology were used to identify GCOC.

**Interventions:**

Considering the recurrence of GCOC and poor effects of single surgery, postoperative adjuvant chemotherapy and concurrent chemoradiotherapy were performed after radical surgical excision.

**Outcomes:**

So far, no significant evidence has suggested recurrence or metastasis after a long-term follow-up.

**Lessons:**

We advocate wide surgery with clean margins at the first accurate diagnosis. Combined modality therapy could be taken for the recurrent GCOC. We expect to provide a new treatment strategy to prevent the growth of this neoplasm.

## 1 Introduction

According to the latest World Health Organization classification in 2005, malignant odontogenic epithelium tumors consist of metastatic ameloblastoma, ameloblastic carcinoma, primary intraosseous carcinoma, ghost cell odontogenic carcinoma (GCOC), and clear cell odontogenic carcinoma.^[1]^ GCOC is defined as a malignant odontogenic epithelial tumor with the features of a calcifying cystic odontogenic tumor, a dentinogenic ghost cell tumor, or both.^[1]^ GCOC has a wide spectrum of biological characteristics.^[2,3]^ Here, we report a case of recurrent maxillary GCOC with suspected cholesterol granuloma of the maxillary sinus (CGMS), which was improperly diagnosed as calcifying epithelial odontogenic tumor (CEOT). We have described the clinical symptoms, radiographic features, histological characteristics, treatment, and follow-up.

## 2 Case report

A 41-year-old Chinese male visited our hospital in 2013, with a 4-month history of bloody purulent rhinorrhea with a peculiar smell in the right nasal cavity. We reviewed the patient's medical history. The patient had been referred to a hospital in Guangzhou in 2008 for a 3-year history of bloody rhinorrhea and nasal obstruction in the right nasal cavity. At that time, physical examination revealed congested mucous, enlarged inferior turbinate, enlarged middle nasal meatus, and markedly impaired sense of smell on the right side. Surgical resection was performed under general anesthesia. The lesion was histopathologically diagnosed as CGMS.

In 2012, nearly 4 years after the first treatment, the patient began to show nasal obstruction again and complained of no alleviation of bloody rhinorrhea since the first operation. The patient went to a hospital in Tianmen for help. According to his medical history and the results of radiological examinations, the patient underwent a radical operation. The pathologic diagnosis was CEOT.

**Figure 1:**
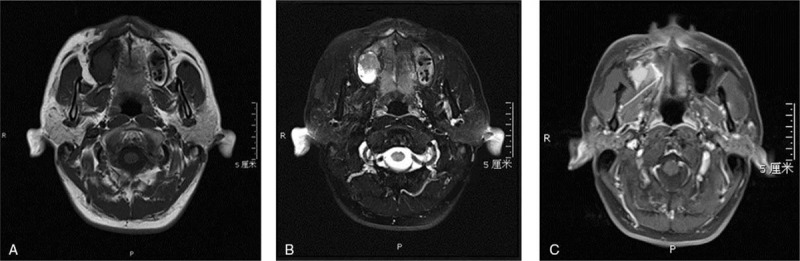
MR showed a soft tissue mass. (A) T1-weighted image (axial). (B) T2-weighted image (axial). (C) Contrast-enhanced T1-weighted image (axial).

**Figure 2:**
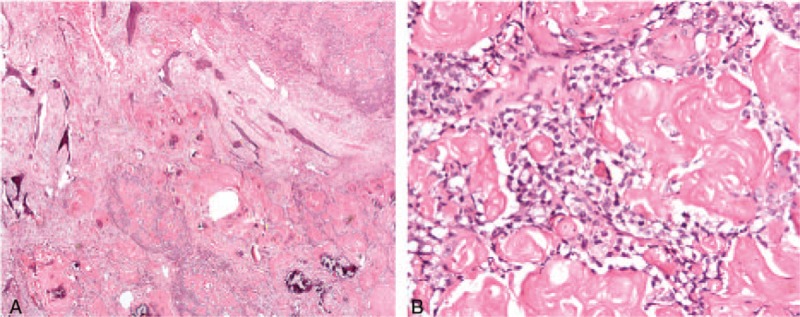
(A) Neoplastic nests are composed of small round cells and ghost cells. Calcification also can be founded (H&E stain, original magnification ×20); (B) Tumor is mixes with 2 kinds of components, which are deeply staining small round cells and ghost cells (H&E stain, original magnification ×200).

In 2013, 1 year after the second operation, the patient came to another hospital in Guangdong with a 4-month symptom of bloody purulent rhinorrhea accompanied by a peculiar smell in the right nasal cavity. It was proved pathologically to be keratinizing squamous cell carcinoma after biopsy. Without any further treatment, the patient came to our hospital in May 14, 2013. Magnetic resonance (MR) imaging revealed a soft tissue mass measuring 3.5 × 2.5 × 2.9 cm located in the right maxillary sinus, which presented mixed, slightly high signal intensity on a T1-weighted image and slightly high signal intensity on a T2-weighted image. This was surrounded by the liquid, high signal intensity on a T2-weighted image, and the lesion invaded all walls of the right maxillary sinus and adjacent zygoma, extending into the nasal cavity and ethmoidal sinus on the right side at the same time. The contrast- enhanced MR showed significant heterogeneous density (Fig. 1). Upon inspection of the emission computed tomography (CT) and lung CT, no evidence supported metastasis. Considering his medical history, we reviewed his hematoxylin and eosin stain slices in 2012. We revised the previous pathologic diagnosis as GCOC. Histopathologically, we observed the neoplastic nests. Parts of the tumor were calcified. The tumor also infiltrated the surrounding connective tissue and bone (Fig. 2A). It was surrounded by the deeply stained small round cells, typical ghost cells in clusters or isolated with pale swollen homogeneous eosinophilic cytoplasm, which had lost their nuclei (Fig. 2B).

Under general anesthesia, a radical surgery was performed. Systemic chemotherapy by intravenous administration of docetaxel (75 mg/m^2^ on day 1) and cisplatin (75 mg/m^2^ on day 1) was carried out on May 31, 2013. After 2 cycles of chemotherapy, the patient received concurrent chemoradiotherapy (planning target volume [PTV_1_] 60 Gy/27F, PTV_2_ 54 Gy/27F, PTV_3_ 50 Gy/27F during weeks 1–5) using a 3-dimensional conformal radiation therapy technique together with 4 weekly docetaxel (40 mg). The adverse effects were decreased appetite, pigmentation of skin in radiation field, and bone marrow suppression, especially thrombocytopenia. At the end of the combined modality therapy, the patient showed good results without any residual neoplasm in radiography. The patient showed a good therapeutic result after the combined modality therapy. No evidence of recurrence or metastasis was observed after the 20-month follow-up, which lasted until May 2015. We will continue to focus on the patient's follow-up.

## 3 Discussion

GCOC is a rare malignant odontogenic tumor that was first described by Ikemura et al.^[4]^ To our knowledge, 36 cases of GCOC have been reported in English-language literature (Table 1).^[4–29]^ Three pathogenic mechanisms explain the origin of GCOC. The first one is that GCOC occurs secondary to calcifying cystic odontogenic tumors. The second is that GCOC is caused by dentinogenic ghost cell tumors. The last one is that it arises de novo.^[3,30]^ A secondary onset from an undiagnosed primary lesion can be potentially considered the de novo type.^[3]^ Owing to a suspected diagnosis as CGMS, this case seemed to fit the last one.

**Table 1:**
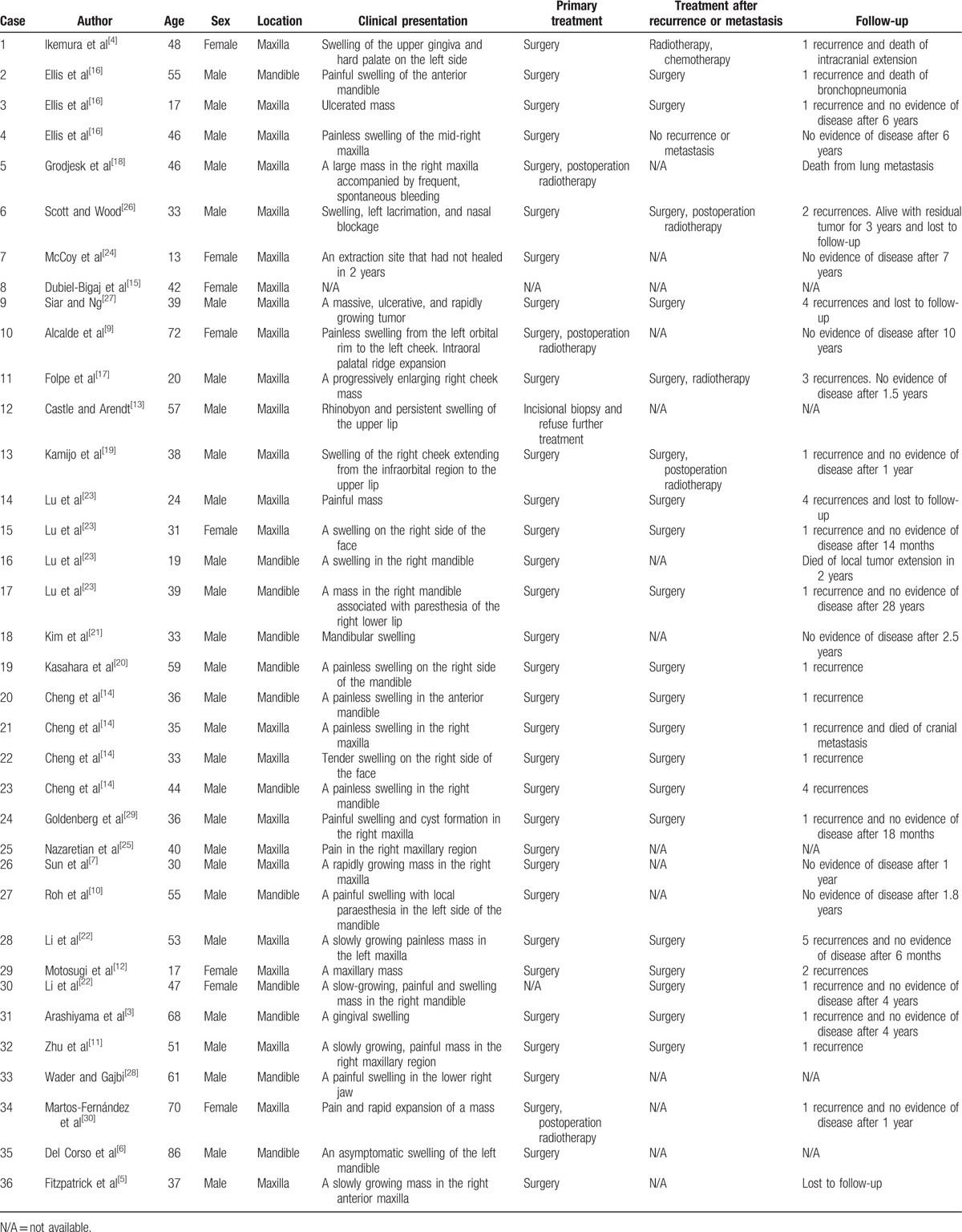
The clinical features of GCOC in English-language literature.

GCOC has a low incidence. In China, malignant odontogenic tumors account for 5.06% of all odontogenic tumors, which accounted for only 0.23% of GCOC.^[31]^ GCOC appears to be more common in Asians and in males.^[6,14,23]^ It can occur at any age with a peak incidence in the fourth decade of life.^[6,14,32]^ GCOC occurs more frequently in the maxilla than the mandible.^[6,14,23]^ The most common clinical symptom of GCOC is a painful swelling in jaws with local paresthesias.^[14]^ The most typical radiological features of GCOC reveal a mixed radiolucent and radiopaque lesion pattern with poorly defined borders, with or without root resorption and tooth displacement.^[14]^ In this article, a 41-year-old Chinese male came to our hospital with a maxillary lesion. The patient showed atypical symptoms of GCOC, such as bloody purulent rhinorrhea and peculiar smell in the right nasal cavity. The age of onset in this case was typical for GCOC. As far as we are concerned, his atypical clinical features may be attributable to the suspected CGMS.

CG is a foreign body reaction to the deposition of cholesterol crystals. It is usually associated with middle ear diseases.^[1]^ It is a rare condition in the maxillary sinus. It was first reported by Graham and Michaels. The clinical features of CGMS are nonspecific. But the most common symptoms are nasal discharge, nasal obstruction and congestion, postnasal drip, facial pain and headache, and otalgia.^[33]^ Although the exact mechanism of the development of CGMS is not clear, Chao^[34]^ proposed probable mechanisms, specifically the obstruction of ventilation, hemorrhage, and impaired drainage. It seems that the appearance of CGMS and GCOC is irrelevant.

In the present case, the patient was diagnosed as CGMS after his first operation in 2008. We suspected this diagnosis may not have been accurate. It seems impossible that 2 kinds of rare tumors would occur in one individual. Because of the limited medical records of the first visit, especially the loss of pathological section, we cannot confirm whether the patient suffered CGMS before GCOC. The patient was diagnosed with CEOT nearly 4 years after his first operation in 2012. By reviewing his pathological section, we finally confirmed that the patient suffered GCOC rather than CEOT. Although GCOC and CEOT are odontogenic origin epithelial tumors producing calcifying materials from transformed epithelial tumor cells, CEOT is a benign odontogenic tumor, considerably different from the malignant GCOC.^[35]^

The diagnosis of odontogenic tumors should not be based solely on clinical symptoms or radiological examination, but must rely on histopathology. Here, we summarize the histopathological features of CGMS, CEOT, and GCOC. CGMS is characterized by a large number of cholesterol clefts surrounded by multinucleated giant cells, histiocytes, plasma cells, lymphocytes, and hemosiderin deposits.^[34]^ CEOT represents retrogressive calcific changes, amyloid-like deposition, and clear cytoplasm.^[1,35]^ Cheng et al^[14]^ put forward histological diagnostic criteria for GCOC, which are an epithelial lining with a well-defined basal layer of columnar cells, a layer of cells resembling the stellate reticulum of the enamel organ, and masses of ghost cells that may be calcified or not, accompanied by atypical epithelial cell foci presenting mitosis, keratin pearls, necrosis, and so on. Some studies also point out that the expressions of immunohistochemical biomarkers of Ki-67 and MMP-9, which are associated with tumor proliferation and invasion, are positive in GCOC.^[36]^ As a result of the high levels of expression of those biomarkers, Ki-67 and MMP-9 can be helpful in the diagnosis and evaluation of the prognosis of GCOC.^[6,11]^ In short, the pathologists should pay more attention to these histopathological features and provide the accurate pathology reports, which is important to the clinical doctors. Immunohistochemistry may help to determine the benign or malignant nature of odontogenic tumors.

The recommended treatment of GCOC is wide surgical excision with clean margins.^[2,3,6]^ The use of postoperative adjuvant chemotherapy and chemoradiotherapy is controversial. Because of the recurrence of GCOC and poor effects of single surgery, we take comprehensive therapy in this case. Two cycles of chemotherapy and chemoradiotherapy together with 4 rounds of weekly chemotherapy after wide surgery were performed. The patient's symptoms showed significant improvement. Meanwhile, cervical lymph nodes were reduced after this therapeutic strategy. The overall 5-year survival rate is estimated to be 73%.^[23]^ The recurrence of GCOC is possible. However, the metastasis is very uncommon. To date, only 2 cases have been reported to cause cranial and pulmonary metastases.^[14,18]^

In summary, GCOC is a rare malignant odontogenic tumor with a high rate of recurrence. We advocate wide surgery with clean margins at the first accurate diagnosis. Regarding recurrent GCOC, we were able to perform combined modality therapy, which combines surgery, postoperative adjuvant chemotherapy, and concurrent chemoradiotherapy. Long-term follow-up is important for observation of recurrence or metastases.

## References

[1] Barnes L Eveson JW Reichart P World Health Organization Classification of Tumours: Pathology and Genetics of Head and Neck Tumours. 2005;Lyon: IARC Press, 283–327; 342–344.

[2] Goldenberg D Sciubba J Tufano RP Odontogenic ghost cell carcinoma. Head Neck 2004;26:378–81. 15054742 10.1002/hed.10376

[3] Arashiyama T Kodama Y Kobayashi T Ghost cell odontogenic carcinoma arising in the background of a benign calcifying cystic odontogenic tumor of the mandible. Oral Surg Oral Med Oral Pathol Oral Radiol 2012;114:e35. 10.1016/j.oooo.2012.01.018 22862988

[4] Ikemura K Horie A Tashiro H Simultaneous occurrence of a calcifying odontogenic cyst and its malignant transformation. Cancer 1985;56:2861. 4052957 10.1002/1097-0142(19851215)56:12<2861::aid-cncr2820561224>3.0.co;2-l

[5] Fitzpatrick SG Hirsch SA Listinsky CM Ameloblastic carcinoma with features of ghost cell odontogenic carcinoma in a patient with suspected Gardner syndrome. Oral Surg Oral Med Oral Pathol Oral Radiol 2015;119:e241. 25434693 10.1016/j.oooo.2014.09.028

[6] Del Corso G Tardio ML Gissi DB Ki-67 and p53 expression in ghost cell odontogenic carcinoma: a case report and literature review. Oral Maxillofac Surg 2015;19:85. 25216652 10.1007/s10006-014-0465-2

[7] Sun ZJ Zhao YF Zhang L Odontogenic ghost cell carcinoma in the maxilla: a case report and literature review. J Oral Maxillofac Surg 2007;65:1820. 17719405 10.1016/j.joms.2006.06.289

[8] Li BH Cho YA Kim SM Recurrent odontogenic ghost cell carcinoma (OGCC) at a reconstructed fibular flap: a case report with immunohistochemical findings. Med Oral Patol Oral Cir Bucal 2011;16:e651. 20729798 10.4317/medoral.17207

[9] Alcalde RE Sasaki A Misaki M Odontogenic ghost cell carcinoma: report of a case and review of the literature. J Oral Maxillofac Surg 1996;54:108. 8530988 10.1016/s0278-2391(96)90317-1

[10] Roh GS Jeon BT Park BW Ghost cell odontogenic carcinoma of the mandible: a case report demonstrating expression of tartrate-resistant acid phosphatase (TRAP) and vitronectin receptor. J Craniomaxillofac Surg 2008;36:419. 18674923 10.1016/j.jcms.2008.06.001

[11] Zhu ZY Chu ZG Chen Y Ghost cell odontogenic carcinoma arising from calcifying cystic odontogenic tumor: a case report. Korean J Pathol 2012;46:478. 23136575 10.4132/KoreanJPathol.2012.46.5.478 PMC3490111

[12] Motosugi U Ogawa I Yoda T Ghost cell odontogenic carcinoma arising in calcifying odontogenic cyst. Ann Diagn Pathol 2009;13:394. 19917476 10.1016/j.anndiagpath.2009.02.008

[13] Castle JT Arendt DM Aggressive (malignant) epithelial odontogenic ghost cell tumor. Ann Diagn Pathol 1999;3:243. 10459050 10.1016/s1092-9134(99)80056-2

[14] Cheng Y Long X Li X Clinical and radiological features of odontogenic ghost cell carcinoma: review of the literature and report of four new cases. Dentomaxillofac Radiol 2004;33:152. 15371314 10.1259/dmfr/67909783

[15] Dubiel-Bigaj M Olszewski E Stachura J The malignant form of calcifying odontogenic cyst. A case report. Patol Pol 1993;44:39. 8488081

[16] Ellis GL Shmookler BM Aggressive (malignant?) epithelial odontogenic ghost cell tumor. Oral Surg Oral Med Oral Pathol 1986;61:471. 3459124 10.1016/0030-4220(86)90390-7

[17] Folpe AL Tsue T Rogerson L Odontogenic ghost cell carcinoma: a case report with immunohistochemical and ultrastructural characterization. J Oral Pathol Med 1998;27:185. 9563575 10.1111/j.1600-0714.1998.tb01938.x

[18] Grodjesk JE Dolinsky HB Schneider LC Odontogenic ghost cell carcinoma. Oral Surg Oral Med Oral Pathol 1987;63:576. 2438624 10.1016/0030-4220(87)90231-3

[19] Kamijo R Miyaoka K Tachikawa T Odontogenic ghost cell carcinoma: report of a case. J Oral Maxillofac Surg 1999;57:1266. 10513878 10.1016/s0278-2391(99)90502-5

[20] Kasahara K Iizuka T Kobayashi I A recurrent case of odontogenic ghost cell tumour of the mandible. Int J Oral Maxillofac Surg 2002;31:684. 12521331 10.1054/ijom.2002.0239

[21] Kim J Lee EH Yook JI Odontogenic ghost cell carcinoma: a case report with reference to the relation between apoptosis and ghost cells. Oral Surg Oral Med Oral Pathol Oral Radiol Endod 2000;90:630. 11077388 10.1067/moe.2000.109016

[22] Li BB Gao Y Ghost cell odontogenic carcinoma transformed from a dentinogenic ghost cell tumor of maxilla after multiple recurrences. Oral Surg Oral Med Oral Pathol Oral Radiol Endod 2009;107:691. 19272810 10.1016/j.tripleo.2009.01.008

[23] Lu Y Mock D Takata T Odontogenic ghost cell carcinoma: report of four new cases and review of the literature. J Oral Pathol Med 1999;28:323. 10432199 10.1111/j.1600-0714.1999.tb02048.x

[24] McCoy BP O’Carroll MK Hall JM Carcinoma arising in a dentinogenic ghost cell tumor. Oral Surg Oral Med Oral Pathol 1992;74:371. 1408002 10.1016/0030-4220(92)90078-5

[25] Nazaretian SP Schenberg ME Simpson I Ghost cell odontogenic carcinoma. Int J Oral Maxillofac Surg 2007;36:455. 17141471 10.1016/j.ijom.2006.10.007

[26] Scott J Wood GD Aggressive calcifying odontogenic cyst—a possible variant of ameloblastoma. Br J Oral Maxillofac Surg 1989;27:53. 2920164 10.1016/0266-4356(89)90127-7

[27] Siar CH Ng KH Aggressive (malignant?) epithelial odontogenic ghost cell tumour of the maxilla. J Laryngol Otol 1994;108:269. 8169519 10.1017/s0022215100126507

[28] Wader J Gajbi N Neoplastic (solid) calcifying ghost cell tumor, intraosseous variant: report of a rare case and review of literature. J Clin Diagn Res 2013;7:1999. 24179921 10.7860/JCDR/2013/6115.3383 PMC3809660

[29] Goldenberg D Sciubba J Tufano RP Odontogenic ghost cell carcinoma. Head Neck 2004;26:378. 15054742 10.1002/hed.10376

[30] Ledesma-Montes C Gorlin RJ Shear M International collaborative study on ghost cell odontogenic tumours: calcifying cystic odontogenic tumour, dentinogenic ghost cell tumour and ghost cell odontogenic carcinoma. J Oral Pathol Med 2008;37:302. 18221328 10.1111/j.1600-0714.2007.00623.x

[31] Luo HY Li TJ Odontogenic tumors: a study of 1309 cases in a Chinese population. Oral Oncol 2009;45:706. 19147397 10.1016/j.oraloncology.2008.11.001

[32] Lu Y Xuan M Takata T Odontogenic tumors. A demographic study of 759 cases in a Chinese population. Oral Surg Oral Med Oral Pathol Oral Radiol Endod 1998;86:707. 9868729 10.1016/s1079-2104(98)90208-6

[33] Karaky AA Sawair FA Baqain ZH Cholesterol granuloma of the maxillary sinus encountered during floor augmentation procedure: a case report. Clin Implant Dent Relat Res 2010;12:249. 19438963 10.1111/j.1708-8208.2009.00151.x

[34] Chao TK Cholesterol granuloma of the maxillary sinus. Eur Arch Otorhinolaryngol 2006;263:592. 16506040 10.1007/s00405-006-0015-0

[35] Lee SK Kim YS Current concepts and occurrence of epithelial odontogenic tumors: II. Calcifying epithelial odontogenic tumor versus ghost cell odontogenic tumors derived from calcifying odontogenic cyst. Korean J Pathol 2014;48:175. 25013415 10.4132/KoreanJPathol.2014.48.3.175 PMC4087130

[36] Gomes da Silva W Ribeiro Bartholomeu dos Santos TC Cabral MG Clinicopathologic analysis and syndecan-1 and Ki-67 expression in calcifying cystic odontogenic tumors, dentinogenic ghost cell tumor, and ghost cell odontogenic carcinoma. Oral Surg Oral Med Oral Pathol Oral Radiol 2014;117:626. 24534622 10.1016/j.oooo.2014.01.021

